# Zero Discharge Performance of an Industrial Pilot-Scale Plant Treating Palm Oil Mill Effluent

**DOI:** 10.1155/2015/617861

**Published:** 2015-01-22

**Authors:** Jin Wang, Qaisar Mahmood, Jiang-Ping Qiu, Yin-Sheng Li, Yoon-Seong Chang, Li-Na Chi, Xu-Dong Li

**Affiliations:** ^1^College of Agriculture and Biology, Shanghai Jiao Tong University, Shanghai 200240, China; ^2^Department of Environmental Sciences, COMSATS Institute of Information Technology, Abbottabad 22060, Pakistan; ^3^Ronser Bio-Tech Sdn Bhd, C708, Metropolitan Square, Bandar Damansara Perdana, 47820 Petaling Jaya, Malaysia; ^4^College of Environmental Science and Engineering, Shanghai Jiao Tong University, Shanghai 200240, China

## Abstract

Palm oil is one of the most important agroindustries in Malaysia. Huge quantities of palm oil mill effluent (POME) pose a great threat to aqueous environment due to its very high COD. To make full use of discharged wastes, the integrated “zero discharge” pilot-scale industrial plant comprising “pretreatment-anaerobic and aerobic process-membrane separation” was continuously operated for 1 year. After pretreatment in the oil separator tank, 55.6% of waste oil in raw POME could be recovered and sold and anaerobically digested through 2 AnaEG reactors followed by a dissolved air flotation (DAF); average COD reduced to about 3587 mg/L, and biogas production was 27.65 times POME injection which was used to generate electricity. The aerobic effluent was settled for 3 h or/and treated in MBR which could remove BOD_3_ (30°C) to less than 20 mg/L as required by Department of Environment of Malaysia. After filtration by UF and RO membrane, all organic compounds and most of the salts were removed; RO permeate could be reused as the boiler feed water. RO concentrate combined with anaerobic surplus sludge could be used as biofertilizer.

## 1. Introduction

Palm oil is used as food as well as biofuel in various non-food-manufacturing industries. Palm oil is one of the most important agroindustries in the tropical regions, notably in Malaysia and Indonesia. Malaysia covered about 5 million hectares of the palm cultivated area with 426 operating mills in 2011 [1]. However, the palm oil production generates a huge amount of palm oil mill effluents (POME). It is estimated that about 1.5 m^3^ of water is required to process 1 ton of fresh fruit bunch (FFB); half of this amount results in POME [2]. POME is a yellow, concentrated liquid with a distinct offensive odor which is characterized by the chemical oxygen demand (COD) and biochemical oxygen demand (BOD) in the range of 44,300–102,696 mg/L and 25,000–65,714 mg/L, respectively. Due to its acidic nature (pH 3.4–5.2), high salt and suspended solids (18,000–46,011 mg/L), and oil and grease, 4000–9341 mg/L [1], the untreated discharge of POME can result in considerable environmental consequences.

The characteristics of POME especially high soluble organic substances render it suitable to be treated through biological means. Anaerobic digestion has widely been accepted as an effective biotechnology for the POME treatment. Anaerobic digestion can be accomplished in a closed-tank anaerobic digester, an open digester tank, or a covered lagoon. Conventional facultative lagoons and open digesting tanks are the most commonly used anaerobic processes for the treatment of POME; although these processes require relatively little operational cost and energy, they demand longer retention times often in excess of 20 days, even 60 days, and large area compared to recent developed treatment methods [2–5]. Additionally, these conventional anaerobic digesters are difficult to collect and utilize the produced biogas, and the biogas mixture containing methane and carbon dioxide produced from open lagoons and tanks directly escaped into the atmosphere. These significantly contribute to the global warming as methane has 21 times more global warming potential than carbon dioxide contributing to a serious global warming problem [3]. The bioconversion of organic waste materials to biogas in anaerobic digesters has practical potential and economic implications. The biogas containing methane is a very promising source of renewable energy which can be harnessed to generate electricity.

On the other hand, anaerobic treatment of POME cannot meet discharge standards due to its high organics content. In view of its energy recovery and environmental conservation, the POME treatment is very critical. The present tertiary treatment technologies can meet the regulatory BOD effluent discharge limits of 100 mg/L under optimum operation. Most of the technologies employed have uncertainties in the plant performance and thus do not consistently meet 100% compliance of BOD (20 mg/L recently proposed) by the Department of Environment (DOE) of Malaysia. In some sensitive regions, especially those involving tourism activities in Sabah and Sarawak, the oil palm industry is required to investigate a wide range of approaches to treat POME.

An innovative approach towards zero-effluent discharge or zero-emissions would enable problem-free mill operation. The main objective is to recover usable materials such as water and oil from the effluent and to minimize the waste generation along the recovery of valuable nutrients from treated sludge which can be reused as biofertilizer. An integrated POME treatment green technology mainly involved in “pretreatment-anaerobic and aerobic-membrane separation” was proposed based on clean development mechanism (CDM) strategy according to Loh et al. [2]. The treatment strategy was tested for 1 year based on 10 hr daily operation. It is imperative to ensure that proposed system is very stable before its industrial application.

To achieve goals like stable high performance of the suggested treatment strategy reduction in hydraulic retention time (HRT), occupied area and greenhouse gases emission of POME, and material recovery from discharged wastes, the present pilot-scale wastewater treatment plant was continuously assessed to evaluate its efficiency. The treatment plant was fed with higher organic loading rate (OLR) and its performance was compared with existing literature.

## 2. Materials and Methods

### 2.1. Experimental Set-Up

**Figure 1:**
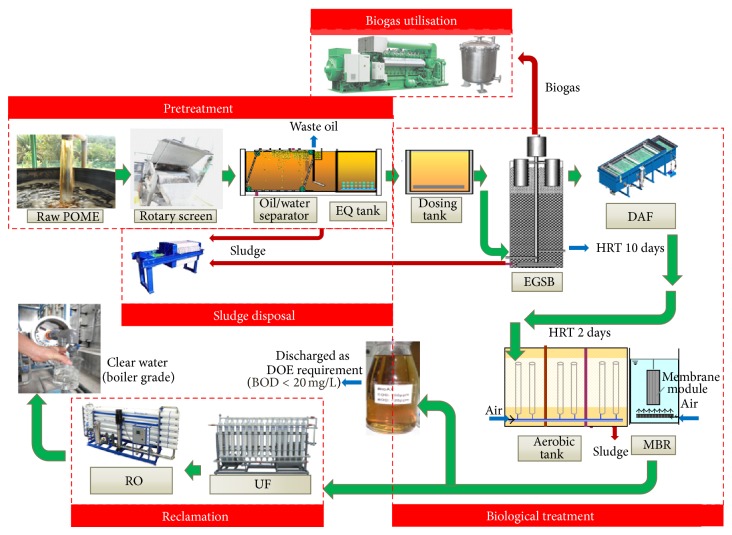
Schematic diagram of palm oil mill effluent treatment plant.

This zero discharge POME pilot-scale treatment plant was installed at Kilang Kelapa Sawit (KKS) Sime Darby Plantation. The schematic diagram of the pilot plant has been shown in Figure 1.

Five main unit processes were included in the system, namely, pretreatment unit, biological treatment unit, reclamation unit, biogas utilisation, and sludge treatment unit. All tanks were made of concrete except for 2 anaerobic expanded granular sludge bed (EGSB) bioreactors made of steel with coating.

The pretreatment unit (Figure 1) comprised rotary screen, grit separator, equalization tank (EQ tank), oil-water separator tank, and cooling tower. The biological treatment unit consisted of two anaerobic EGSB steel tanks, each with diameter and height of 6 m and 16 m, respectively, with the total volume of 423.9 m^3^. The reactor walls were covered with cloth acting as insulation layer to preserve heat. The reactor was operated at constant temperature (average 35°C); further, the cooling tower helped to control the influent temperature. EGSB tanks were designed for running in parallel in this study; other tanks included 2 dosing tanks, a dissolved air flotation (DAF) set, a biocontact aerobic tank, and a membrane bioreactor (MBR). The reclamation unit comprised two modules of ultrafiltration (UF) membrane (LH3-1060-V) containing nominal molecular weight cut-off (MWCO) of 50,000 Dalton (Litree, China). Another module was PROC-10 reverse osmosis (RO) membrane (Hydranautics, USA) having 99.7% NaCl rejection rate. For the protection of the membrane modules, a safety filter was installed between MBR and UF; another fine filter was located between UF and RO. Biogas purifier (containing Fe_2_O_3_ for H_2_S, CO_2_, and moisture removal) and a generator were used to transform biogas into electrical energy. The surplus sludge mainly from EGSB system and the pretreatment unit was piped and condensed in sludge tank and then dewatered by the sludge frame filter press. The retentate from RO and the surplus sludge from anaerobic unit were meant for land farming.

### 2.2. Characteristics of the Feed POME

The open lagoon system in KKS Labu, Sime Darby, comprised a cooling pond, an acidification pond, 2 anaerobic ponds, 2 facultative ponds, and a final discharge pond. With the current processing capacity of the mill, the overall HRT of this lagoon system is above 100 days. Loh et al. [2] presented the operational performance during the first year of POME treatment plant based on 10 hr daily operation. The system was operated continuously for 24 hr for another year. The influent was pumped from cooling pond and the sampling points were marked less than 1 km from it.

The characteristics and variations in POME from different sources were summarized in Table 1. The POME under investigation (source c) had higher COD concentration compared to the other normal POME as given in Table 1.

### 2.3. Analytical Methods

Various parameters of water quality like pH, COD, SS (suspended solids), VSS (volatile suspended solids), VFA (volatile fatty acid), total alkalinity, color, odor, turbidity, specific conductance, TDS (total dissolved solids), and total hardness were analyzed according to the standard methods for examination of water and wastewater [7].

BOD_3_ at 30°C was analyzed based on the method developed by DOE [8]. Heavy metals were analyzed by the inductively coupled plasma atomic emission spectrometry (ICP-AES) using an Iris Advantage 1000 spectroscope (Thermo Jarrell Ash Co., USA). Raw POME was pretreated in aqua regia. The microscopic features of the anaerobic granular sludge were investigated in a scanning electron microscope (SIRION 200, FEI, USA) at 5 kV. Oil and grease (O and G) content was determined according to the method of Zhang et al. [5].

## 3. Results and Discussion

In 2011, the pilot plant system was operated intermittently and operated for 10 hr daily. To achieve higher efficiency towards pollutant-free zero discharge, the POME treatment plant was operated for another year during which plant worked for 24 hr daily. During this stage, some important parameters were adjusted.

### 3.1. Pretreatment Performance

**Figure 2:**
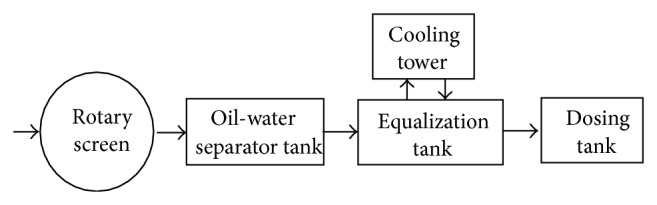
The schematic diagram of pretreatment process.

The schematic diagram of pretreatment process was shown in Figure 2. POME contained high O and G, SS, and temperature. O and G and SS are generally considered as an obstacle for an efficient biological treatment, while most SS can be anaerobically biodegraded at longer HRT. The excessive surface scum could choke the biogas outlet resulting in spilling of contents, even granular sludge. The formation of scum is normally attributed to the presence of O and G in the raw POME; however, no pretreatment was administered before entry to the anaerobic reactor [9]. The removal of O and G from POME was considered essential to guarantee its effective treatment and the recovery of O and G can be a byproduct for reutilization.

The gross solids and unexpected mass in the raw POME were removed by a rotary screen prior to oil-water separation. The removal of gross pollutants from POME protects downstream equipment, avoids interference with plant operations, and prevents the entry of objectionable floating material into the system, especially anaerobic tanks. Considering the presence of sand in POME, the grit separator was adopted. The rotary screen was put on the grit separator. An EQ tank was used as a buffer to stabilize the operational parameters such as flow, pollutant concentration levels, and temperature. Prior to EQ tank, the oil separating tank was used to collect sludge oil. To reduce the temperature of the influent of anaerobic process to the optimum mesophilic range (35 ± 2°C), a cooling tower was installed. The dosing tank adjusted the pH to neutral (about 7). However, there was no need to dose caustic to adjust pH during the operation of POME treatment.

The pretreatment unit was effective enough to bring a decrease in the concentrations of COD and O and G as compared to the raw POME as shown in Table 2. The average COD concentration of EQ tank effluent was 71179 mg/L.

### 3.2. Performance of the Biological Treatment

The anaerobic EGSB process played significant role in degrading the bulk of organic content. The effluent SS were removed by DAF whose effluent discharged to the attached growth aerobic tank. The characteristics of treated samples from each biological treatment unit were shown in Table 3.

**Figure 3:**
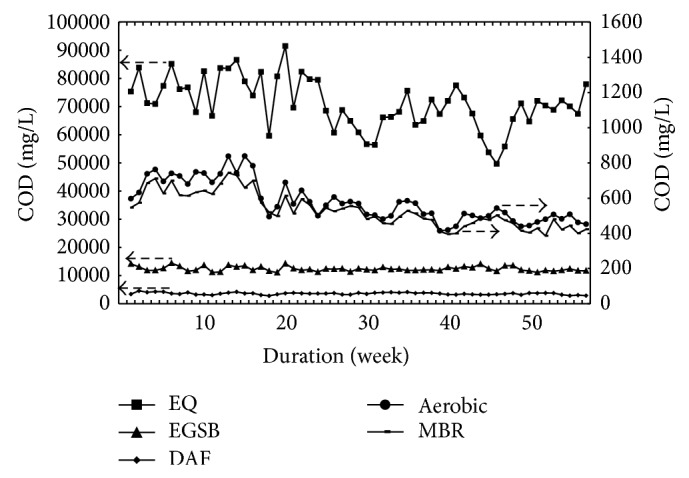
The variations of influent and effluent COD concentration.

The variations in the influent and effluent COD concentration for biological units including anaerobic EGSB, DAF, aerobic tank effluent (settled 3 hr), and MBR effluent were presented in Figure 3.

The anaerobic EGSB reactors reduced the amount of SS to 20–40 g/L (15–30 g VSS/L) after the intermittent operation for one year. The reactors operated at HRT of 9.8 d. Table 3 and Figure 3 suggested an overwhelming COD reduction during anaerobic EGSB operation. On average, COD decreased from 71179 to 12341 mg/L, after SS removal in DAF. The average COD concentration was 3587 mg/L with COD removal rate of 94.89%. As half of the sludge from DAF was recycled to EGSB tank, which implied that half of the recycled sludge was biodegraded during anaerobic treatment, thus actual COD removal was 88.65%.

Anaerobic digestion is a well-established wastewater treatment technology. Sludge granulation is considered to be the most critical parameter affecting successful operation of an upflow anaerobic sludge blanket (UASB) and EGSB reactor [10]. The EGSB reactors were seeded with anaerobic flocculent sludge from the local POME treatment plant. Prior to the present study, the system was operated under semicontinuous mode based on 10 hr daily operation for 1 year [2]. Sludge granulation did not occur even after the continuous operation of one year.

During the first 17 weeks of POME treatment, the dosed cationic polymer (PAM) concentration in DAF unit was only about 15 mg/L with the average VFA content of 649.9 mg/L. Though half of the sludge was recycled to the dosing tanks, aerobic average effluent COD was 701.5 mg/L. Subsequently, dosed PAM concentration in DAF unit increased to about 30 mg/L with the average VFA reduction to 476 mg/L. Keeping recycling half of the sludge in EGSB, aerobic average effluent COD was 512 mg/L. The granulation of anaerobic sludge was found after 23rd week. When operated for 57 weeks, bulk of anaerobic sludge was granular in EGSB reactors. It implied that despite high SS, appropriate dose of cationic PAM could accelerate the granulation of anaerobic sludge.

Basri et al. [11] observed biomass washout of 500 m^3^ digester during anaerobic treatment of POME which was caused by the continuous recirculation of effluent. It was recommended that the mixing pump should be stopped at least 2-3 h prior to sludge settling in order to reduce biomass washout. Thus, an appropriate mixing intensity in reactor is crucial for anaerobic treatment of POME.

In order to provide enough oxygen and get enough contact between microbes and substrates, to reduce resistance to mass transfer, the air-liquid ratio (A/L) should be kept at about 15–25 for aerobic industrial wastewater treatment [12, 13]. During the present study, the produced biogas was 27.65 times POME flow rate (see Table 4); thus, the biogas was enough to act as the “mixer” like aeration in aerobic process. Additionally, there was influent distributor along influent nozzle [14, 15] at the bottom of EGSB reactor.

The total alkalinity (as CaCO_3_) of EGSB effluent averaged about 5448 mg/L which helped to maintain the system pH above 6.8 without dosing extra caustic as EGSB was plug flow. Final effluent VFA of this pilot EGSB was about 537 mg/L (as acetic acid) indicating a stable methane production.

High biogas production potential of organic SS renders the process economics more favorable. The pretreatment prior to anaerobic treatment may generate more surplus sludge requiring disposal at additional cost. The hydrolysis SS can be considered as the rate-limiting step during anaerobic treatment of POME. In comparison to previous studies, the present strategy did not help to remove SS from raw POME, rather than prolong and keep the HRT stable at 9.8 d. Three-phrase separator of EGSB helped to settle most of sludge back to reactor. PAM was dosed in DAF unit to improve SS removal rate. SS containing inert substrate ingredients and active biomass from DAF were recirculated to anaerobic unit which increased suspended solids “HRT” in EGSB reactor. The strategy could increase the biodegrade ratio of POME SS to overcome their accumulation and helped to increase the methane yield. At the same time, appropriate PAM dosage accelerated anaerobic sludge granulation due to very high SS.

**Figure 4:**
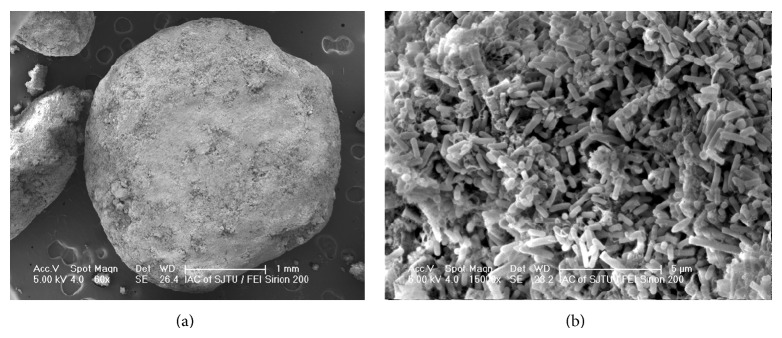
Scanning electron micrographs (SEM) of granular sludge of the EGSB: (a) morphology of anaerobic granules (60x magnification); (b) inner structure of anaerobic granule (15,000x magnification).

The scanning electron micrograph (SEM) of the granular sludge from EGSB reactors was presented in Figure 4. The granules had an average diameter of 1–3.5 mm; bacilli-like bacteria were the dominant microorganisms in the granular sludge.

**Figure 5:**
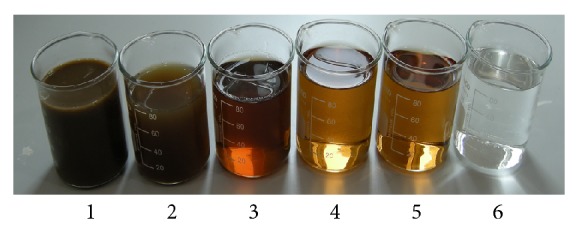
Photograph of all effluent samples from all main units of the POME pilot plant. Note: sample (1) raw POME; (2) anaerobic EGSB+DAF outlet; (3) settled 3 h of aerobic outlet; (4) MBR outlet; (5) UF outlet; (6) RO outlet.

The aerobic process was operated at HRT of 48 h and DO was controlled at about 3.5 mg/L. The effluent COD of biocontact aerobic tank was 579 mg/L after settling for 3 h. To accomplish more stable BOD removal, the aerobic effluent was directly treated in MBR whose final effluent COD was 530 mg/L. It is evident from Figure 5 that MBR accomplished decolourisation partly; however, COD were only reduced 49 mg/L. Both BOD were less than 20 mg/L, achieving the discharge standard set by the Department of Environment (DOE) of Malaysia.

### 3.3. Biogas Production and Utilization

Loh et al. [2] stated that the EGSB produced biogas volume of 52.7 m^3^/h, at a rate of 15–21 m^3^ per m^3^ POME. In this study, the anaerobic EGSB performance parameters and biogas production were presented in Table 4. The produced biogas was about 27.65 m^3^ biogas per m^3^ POME. It approximated to about 28 times based on laboratory studies [16]. The average biogas production efficiency was 0.44 m^3^ biogas/kg COD. The biogas had a rather stable composition of 65–70% CH_4_, 25–36% of CO_2_, and 800–1500 ppm of H_2_S. The methane yield ranged from 0.29 to 0.31 m^3^ biogas (STP)/kg COD obtained in this study which suggested the COD recovery as methane accounted for 82.9–88.6% of theoretical value of 0.35 m^3^  biogas (STP)/kg COD. It could be compared with previous researches [3, 17, 18].

A desulphurization system containing ferric oxide obtained H_2_S removal rate of above 70%. Online Multichannel Gas Analyser Biogas 401 (ADOS Gmbh, Germany) was employed to monitor the biogas composition. H_2_S content of biogas from desulfurization unit should be less than 200 ppm. The produced biogas was used to generate electricity after desulphurization. Most of the produced electricity was sold except that which was partly used in the pilot plant.

### 3.4. Membrane Filtration System

The treated water after biological treatment units was passed through a water reclamation system including UF and RO. Six effluent samples from various POME treatment units were shown in Figure 5. It was evident that MBR and UF effluents had almost the same colors; these effluents could be regarded as the pretreatment of RO. As aerobic effluents after settling period of 3 h could consistently meet 100% compliance for BOD_3_ 20 mg/L, then aerobic treatment alone was sufficient for future industrial application if the aim was to achieve BOD_3_ standard; however, for zero pollutant discharge, MBR can be adopted if rather expensive investment cost is not an issue. The conventional secondary clarifier can be used to remove SS of aerobic effluent.

The zero discharge system used MBR and UF as the pretreatment of RO. After biological treatment, the residual macromolecules in the treated water were removed through MBR and UF before removal of salt ions through RO. UF was operated up to maximum pressure of 3 bars. The transmembrane pressure for UF was maintained at 2 bars by adjusting the pressure control valves. After passing through the fine filter, the collected permeate from the UF unit was fed into the RO unit to produce permeate of boiler grade water standards. RO membrane with 99.7% NaCl retention was operated up to a maximum pressure of 15 bars. The transmembrane pressure for RO was maintained at 12 bars by adjusting the pressure control valves.

When the transmembrane pressure increased to 1.5 times the initial pressure or filtration flux reduced to 90% of the initial flux during each experiment, the UF and RO membranes would be cleaned with chemical solution mixture of 1% (W/W) NaOH and 0.6% (W/W) NaClO for 25 min after first circulation with clean water to flush out POME retained in membranes. The membranes were then rinsed with clean water followed by circulation with 1% (W/W) nitric acid for 25 min to avoid inorganic fouling. All membranes were flushed with clean water to remove the residual nitric acid by monitoring the flush water pH value until a neutral pH was achieved.

RO removed almost all residual large molecules and most of ions from the treated water. RO retentate accounted 36% recovery of rejected water collected as a liquid fertilizer with high potassium content of around 64% of RO permeate (boiler grade) and could be used in the boiler and cooling tower.

In appearance, the treated effluent seemed crystal clear like tap water as shown in Figure 5. MBR/UF membrane unit removed most of the suspended solids and reduced turbidity to 3.2 NTU. However, UF separation could not remove dissolved solids. After RO membrane filtration, organic matter was completely removed, and total organic carbon (TOC) reduced from 193 mg/L of UF permeate to 0.3 mg/L of RO permeate. Odor and O and G were undetectable in RO permeate, and the metal elements were undetectable or found as traces (except for K and Mg). Comparison of the characteristics of RO permeate to the boiler feed water standard set by American Boiler Manufacturers Association (ABMA) [19] given in Table 4 indicated that the RO permeate was good enough to be used as the boiler feed water except for DO. For boiler, the most harmful of the dissolved gases was oxygen. Even every low oxygen concentration can cause severe damage. The oxygen can be removed both mechanically and chemically. The mechanical methods for removing oxygen include the deaerators and vacuum degasifiers. Any remaining oxygen is dealt with by the addition of chemical oxygen scavenger such as catalyzed sodium sulphite. Typically, 8 mg/L of sodium sulphite is sufficient to deal with 1 mg/L of dissolved oxygen. So the RO permeate could be used as boiler feed water combined with conventional unit to remove oxygen.

Table 5 shows all listed indices of the RO permeate were well below the maximum contaminant level (MCL) for the drinking water standard set by US Environmental Protection Agency [20, 21]. TOC value of 0.3 mg/L in the RO permeate was due to the presence of trace amounts of dissolved organic matter. Further analysis of the trace organic compounds of the RO permeate should be conducted to ensure the drinking water safety.

### 3.5. RO Concentrate and Sludge Recovery as Biofertilizer

Fermented POME could enhance maize crop production and leaves considerable level of soil organic carbon, N, and P residues than plots that received no amendment (control), which promoted sustainable agriculture [22]. Nitrification of POME improved the POME quality as a source of liquid nitrogen fertilizer because ammonia liberation and nitrate were more easily absorbable by most plants, especially in oil palm plantations [23].

RO concentrate was fermented POME and contained about 3 times the concentration of liquid potassium (K) compared to raw POME. Potassium (K) is necessary as a conventional fertilizer. RO concentrate also contained natural higher percentage of N, P, and Mg than raw POME. Based on above reports, RO concentrate could be used as liquid fertilizer.

In addition, the surplus sludge from anaerobic reactors could be recovered as biofertilizer which showed better fertilizer values compared to the raw POME. The pot trials showed that the application of organic fertilizers derived from the surplus sludge could enhance the soil fertilization much better than the fertilizer derived from raw POME or from poultry manure [2].

Biofertilizers have been regarded as an alternative to chemical fertilizers to increase soil fertility and crop production in sustainable farming. These are the products containing living cells of different types of functional microorganisms, which have the ability to convert nutritionally important elements from unavailable to available forms through biological processes [24].

Falodun et al. [25] found that soybean responded to POME at 5 and 10 t ha^−1^ while NPK fertilization at 200 kg ha^−1^ resulted in significant increase in grain yield from 1416 to 3213 kg ha^−1^. Sole inorganic fertilizer application resulted in higher vegetative growth than POME. Total dry matter, relative yield, relative agronomic effectiveness, and chlorophyll content indicated that the addition of POME and NPK fertilizer had more significant influence than the control. Integration of 5 t POME and 150 kg NPK ha^−1^ was the optimum treatment combination alternative to sole use of inorganic fertilizer [26]. From above, the anaerobic surplus sludge combined with RO concentrate can be used as biofertilizer.

## 4. Conclusions

The following conclusions can be drawn from the present study.

The pretreatment was necessary for O and G removal ratio of 55.6%. The anaerobic EGSB reactors at HRT of 9.8 d reduced COD on average from 71179 to 12341 mg/L. After SS removal by DAF, the average COD was 3587 mg/L with a removal rate of 94.89%. Bacilli-like bacteria were the dominant microorganisms in the produced granular sludge. The produced biogas was about 27.65 m^3^ per m^3^ POME. The biogas had stable composition of 65–70% CH_4_, 25–36% of CO_2_, and 800–1500 ppm of H_2_S. The produced biogas was employed in electricity generation after desulphurization.

The aerobic reactor was operated at HRT of 48 h and DO concentration of about 3.5 mg/L, and the aerobic effluent after being settled for 3 h contained COD content of 579 mg/L. MBR effluent COD was 530 mg/L, while both BOD were less 20 mg/L.

## Figures and Tables

**Table 1:** Characteristics and composition of raw POME.

Parameter	Source a	Source b	Source c
Temperature (°C)	80–90	ND	ND
pH	4.2	4.15–4.45	4.5 ± 1.19
Chemical oxygen demand (COD)	51000	45500–65000	76896 ± 119
Biochemical oxygen demand (BOD_3_) at 30°C	25000	21500–28500	27500 ± 100
Oil and grease (O and G)	6000	1077–7582	ND
Suspended solids (SS)	18000	15660–23560	27000 ± 82
Ammoniacal nitrogen (NH_3_-N)	35	ND	36 ± 1
Total Kjeldahl nitrogen (TKN)	750	500–800	60 ± 6
Potassium (K)	2270	1281–1928	1154.8 ± 3.14
Magnesium (Mg)	615	254–344	287.8 ± 8.41
Calcium (Ca)	439	276–405	286 ± 4.39
Zinc (Zn)	2.3	1.2–1.8	1.98 ± 0.74
Iron (Fe)	46.5	75–164	65.7 ± 1.09
Copper (Cu)	0.89	0.8–1.6	0.85 ± 0.05

Note: Source a: [6];

Source b: [4];

Source c: [2].

The units of all measured parameters were in mg/L, except pH and temperature. ND: not detected. Values represent means of all determinations ± SD (standard deviation).

**Table 2:** The characteristics of POME after the pretreatment.

Index	Raw influent	After oil-water separator tank	Average removal rate
Oil and grease (mg/L)	9023 ± 1104	4007 ± 704	55.6%
COD (mg/L)	79874 ± 9642	71179 ± 8811	10.9%

Note: all data are shown as means ± standard deviation of all samples.

**Table 3:** Characteristics of POME samples from each biological treatment unit.

Index (mg/L)	EQ tank	EGSB effluent	DAF effluent	Aerobic effluent	MBR
BOD_3_	30314 ± 1803	3564 ± 704	1335 ± 107	16 ± 4	13 ± 5
COD	71179 ± 8811	12341 ± 843	3587 ± 379	579 ± 112	530 ± 95
Suspended solids	32406 ± 2734	11456 ± 2734	1154 ± 82	62 ± 5	ND
Volatile fatty acid (as acetic acid)	/	537 ± 128	/	/	/
Total alkalinity (as CaCO_3_)	/	5448 ± 229	/	/	/

Note: all data were shown as means ± standard deviation of all samples. ND: not detectable; /: not provided.

**Table 4:** Evaluation of anaerobic EGSB in biogas production in the pilot plant.

Parameter	Unit	Average value
2-EGSB total effective volume	m^3^	847.8
Capacity	m^3^/d	86.4 ± 4.1
Influent COD	mg/L	71179 ± 10950
EGSB effluent COD	mg/L	12341 ± 1338
DAF effluent COD	mg/L	3587 ± 546
EGSB effluent COD deducing recycled sludge	mg/L	7917 ± 955
Apparent COD removal rate	%	94.84 ± 1.08
Real COD removal rate	%	88.56 ± 1.97
Biogas production	m^3^/d	2389.0 ± 201.3
COD reduction	kg/d	5465.8 ± 259.4
Organic loading rate (OLR)	Kg COD/m^3^·d	6.45 ± 0.61
Efficiency (in POME injection)	m^3^ biogas/m^3^ POME	27.65 ± 3.02
Efficiency (in POME injection)	m^3^ biogas/kg COD	0.44 ± 0.04

Note: all data are shown as means ± standard deviation of all samples.

**Table 5:** Effluent characteristics of reclamation system comparing standards of boiler feed water and drinking water.

Parameter	Ultrafiltration (UF)	Reverse osmosis (RO)	Boiler feed water standard (Drum pressure 31.1–41.1 bar)	Drinking water standard
pH	8.61	7.53	7.5–10.0	6.5–8.5
Color, color units	85	1	NR	15
Odor, threshold odor number	32	ND	NR	3
Turbidity, NTU	3.2	0.2	NR	<0.5
Specific conductance, *μ*S/cm	4792	295	2500	NR
Total dissolved solids (TDS), mg/L	3389	307	NR	500
Silica, mg/L (SiO_2_)	1.42	0.95	40	NR
Total alkalinity, mg/L (CaCO_3_)	1926	96	250	NR
Oil and grease (O and G), mg/L	ND	ND	0.5	0.3
DO, mg/L	1.76	1.62	0.007	NR
Total hardness, mg/L (CaCO_3_)	792	0.15	0.2	NR
Total organic carbon (TOC), mg/L	193	0.3	0.5	NR
Al, mg/L	ND	ND	0.1	0.05–0.2
K, mg/L	1983	59	NR	NR
Mg, mg/L	164	0.04	0.25	150
Ca, mg/L	39	ND	NR	NR
Fe, mg/L	0.99	ND	0.03	0.3
Mn, mg/L	0.32	ND	0.1	0.05
Cu, mg/L	0.01	ND	0.02	1.0
Zn, mg/L	ND	ND	NR	5

Note: ND: not detectable; NR: not required; the transmembrane pressures (TMP) for UF and RO membrane were maintained at 2 bars and 13 bars, respectively.
